# Understanding Class in the Post-Industrial Era - Thoughts on Modes of Investigation

**DOI:** 10.3389/fsoc.2020.00039

**Published:** 2020-05-28

**Authors:** David S. Byrne

**Affiliations:** Department of Sociology, Durham University, Durham, United Kingdom

**Keywords:** class, post-industrial, methods, place, state, work, dynamism

## Abstract

The essential character of social science is that is founded around an interaction between theoretical framings and empirical investigation. Class is one of the most salient framing concepts of the discipline, always central even if somewhat pushed into the background in an era when identities not founded in economic relations seemed to take priority. Recently in an era of austerity and economic crisis class is very much back front and center. The issue addressed in this article is how can and should we investigate class in the societies in which classes emerged from industrial systems when those societies are now not only post-industrial but also to an increasing degree post-welfare in consequence of the retreat of a coherent class based politics. Traditionally there has been a distinction between studies which used primarily qualitative styles—ethnographic and at the interface of history and sociology—on the one hand and quantitative studies based on the analyses of large data sets on the other. It has to be said that many of the latter used linear modeling approaches which were of questionable value, particularly when the only dynamic element in them was the exploration of just two time points in the life course in relation to social mobility. What will be proposed here is the value not only of studies which deploy ethnographic/historical and related qualitative modes but also of quantitative work, including in particular quantitative histories, which break with the linear model and deploy dynamic modes of investigation across a range of social scales from the individual life course to the whole global social order. One central proposition in the article is that in order to grasp the nature and potential of class after industry we have to engage in *meta-interpretation*—that is scholarly, we might say hermeneutic, reflection on multiple studies conducted in all appropriate modes of social investigation.

## Introduction

This article is written in the frame of complex realism, that synthesis of critical realism as a philosophical ontology and complexity theory as a scientific ontology proposed by Reed and Harvey (1992). A primary task is the establishment of the context in which the discussion is conducted—context defined and delimited in terms of time and space. The focus is on what used to fifty years ago be called the “Advanced Industrial Societies”—that is on the set of nation states which had economies dominated by the industrial production of material commodities and which generally had some form of welfare capitalist organization of state activity. This set included most of Western Europe, Canada, the USA, Australia, and New Zealand. There were some outliers—e.g., the Republic of Ireland which was not predominantly industrial and states of Southern Europe were less industrial at least in part than say the UK or West Germany—but this was the predominant form of the core capitalist democracies of the North. By the 1970s many of the states in the European Soviet system including the Slav republics of the USSR, Poland, Czechoslovakia, East Germany, and Hungary had much in common with this set. In all of these countries both the proportion of the population engaged in or dependent on wages from industrial work and the proportion of economic output (Gross Value Added) generated from industrial activity has declined dramatically. In the UK which in the middle 1960s was probably the most industrialized society there has even been the proportion of the workforce engaged in industrial production broadly defined declined from 45% in 1970 to 18% in 2015. Elsewhere deindustrialization was not so severe but even unified Germany, the industrial powerhouse of the European Union, now has 28% of its workforce in industrial jobs in 2015 compared with 41% in West Germany in 1970 and almost certainly a higher figure in the then East Germany. So industrial employment and the mechanisms through which class was lived in an industrial society no longer predominate in the old heartlands of the industrial working class. There is still an enormous global industrial working class but it is now predominantly located in East and South Asia and other parts of the global south.

The formerly advanced industrial countries continue to have economies organized on the basis of market capitalism and that is now also unequivocally the case for the former soviet system European states. So what exists in these places now is post-industrial capitalism. In realist terms the generative mechanism for the social order remains capitalism with a central element being the wage labor relation. We should also note that the kind of Keynes/Beveridge forms of economic and social policies—in Germany described by the ordo-liberal term the social market economy—which constituted what we might call “welfare capitalism” have also gone into retreat across post-industrial capitalism. Austerity and privatization, most notably in the UK but also in former Soviet style states, have made severe inroads into welfare provision and—this is very important—have changed the nature of employment in health, education and welfare particularly but not only for professional groups in those areas. “The New Public Sector Management” has largely eliminated professional autonomy. Coupled with privatization this has considerably eroded the working conditions and remuneration of many welfare state workers. Things are not what they were. So how do we as social scientists engage with understanding what is going on through empirical social research?

The first necessary element in that programme of understanding derives from the complexity element in the complex realist framing. We need to think in terms of interwoven systems operating at a range of different levels from the global to the single individual and recognize that these systems are dynamic. They can and do change—they have trajectories through time and space—and that grasping and representing the character of the system trajectories is one of the crucial tasks of the social scientist engaged in empirical work. These systems are complex which means that they have emergent properties—they cannot be understood properly by a reductionist programme of explanation in terms of their components. That complex character means that methods of representation which assign causal powers to models constructed from variables are mostly useless. They are dynamic and change but as complex systems generally do not change incrementally but rather by changes of kind rather than degree. They are non-linear. We require modes of representation and exploration of causality which can cope with non-linearity.

And they are interwoven. The word interwoven is a somewhat better description of the relationships among the complex systems of the social—of individuals with households, of both individuals and households with local spatial systems at every level from the neighborhood to the nation state and beyond, of the state as a system with many aspects of individual and household life courses, of all social systems with the generative implications of the capitalist mode of production AND of all social systems with the ecological and natural systems of the planet on which humans live. – than the more commonly employed intersected. Interwoven describes a more inter-related style of relationship among multiple systems than intersection because systems are mixed at every level and have potential causal powers in every direction. All complex systems can be regarded as cases and methods which start from cases as the real objects addressed by scientific inquiry are the appropriate way to engage with them. That means that a range of approaches from case based quantitative methods such as the generation of numerical taxonomies (clustering) and Qualitative Comparative Analysis (QCA) through the intensive ideographic and ethnographic exploration of particular social contexts and systems of relationships are appropriate but other approaches, particularly those which abstract “variables” from the cases themselves, are not.

## The Role of Quantitative Research in Understanding Class

“It is very striking that the classic technique developed in response to the impossibility of understanding contemporary society from experience, the statistical mode of analysis, had its precise origins within the period of (early nineteenth century) of which you are speaking. For without the combination of statistical theory, which in a sense was already mathematically present, and the arrangements for the collections of statistical data, symbolized by the founding of the Manchester Statistical Society, the society that was emerging out of the industrial revolution was literally unknowable.” (Williams, 1980, p. 170).

Williams should have said description rather than analysis and statistical theory in the sense of probability based sampling theory had little to do with that description then and only contributes to it now on the basis of inference from samples but the point about the centrality of statistical measures as the mode through which we can see the general trajectory of social systems is absolutely and fundamentally correct. The data on the shift from societies in which class relations were founded on the centrality of experience in industrial production of material commodities to the post-industrial present constitutes precisely such a description of trajectory and radical change. At that level we dealt with class in relation to the productive systems of nation states but people are what matter. (Thompson, 1978, p. 85) famously and correctly asserted that class is not a thing, it is formed through relationships and manifests as a happening. That said we can use measures, understood as Byrne (2002) has argued not as variables with causal powers which can be abstracted from the real cases but rather as trace descriptions of those cases at points through time, to say something about the nature of the entities at multiple levels through which class relationships are actually constituted.

Let us start with individuals. The conventional mode of assigning class as an attribute to individuals is on some sort of occupationally derived basis. Generally insofar as this is justified by any connection to a broad theoretical framing of class, this is done by reference to Weber and based on the contention that occupations are relatively stable throughout a life course and therefore are a better guide to life chances than say incomes which can change radically over short time periods. Rose and Pevalin (2003) go so far as to assert that income is merely epiphenomenal to occupation. If it was ever the case that occupations were stable through a life course, and there is considerable evidence that this could be challenged even in the era of industrial capitalism, then in post-industrial capitalism this is manifestly not true. Moreover, in societies where the great majority of women of working age in couple relationships are in paid employment the significant unit for income and even more for wealth, particularly housing wealth, is the household, not the individual. Individual occupation is a relatively poor indicator of household income, even at a single time point and it is not a good indicator of household wealth. Of course individuals move through households in a life course. That is why panel studies based on households have to use individuals as the constant cases across the waves at which data is collected. Certainly material circumstances as measured by income and wealth matter when we are exploring class and they matter in dynamic terms and need to be described as trajectories. The emphasis on occupation as the attribute through which class can be assigned is fatuous. Of course occupation is one aspect of assigning class and certainly we need to take note of it but the significance of occupation for class as a relation can and does change.

We can see this very clearly if we consider the changing nature of the work relationships of what Goldthorpe has called “the service class” differentiated from a working class and an intermediate class.

“the salariat (or service class) of professional and managerial employees are associated with the regulation of employment via a “service relationship”: i.e., a contractual exchange of a relatively long-term and diffuse kind in which compensation for service to the employing organization comprises a salary and various perquisites and also important prospective elements—salary increments, expectations of continuity of employment (or at least of employability) and promotion and career opportunities. The service relationship will most fully realized with higher-level professionals and managers (Classes I/1), while modified forms will be most common with lower-level professionals and managers (Classes II/2).” (Goldthorpe and McKnight, 2004, p. 5).

In a book published in 2017 (!) (Evans and Tilley, 2017, p. 4) asserted that: “Compared with the working class, middle class workers occupy relatively secure salaried positions, often with occupational pensions and other benefits.” Really? Under the impact of the new public sector management even in the public sector senior professionals are subjected to intense control and work discipline, and this is not least true of academics. Public sector pensions are being cut and the final salary schemes which offered security on retirement have been almost eliminated from the private sector. Things change! Many UK academics, most of those delivering the bulk of undergraduate teaching, are temporary employees at best and many have 0 h contracts. Occupation matters but, in terms of day to day lived experience, so do wealth and income and these are attributes of households rather than individuals.

Let me illustrate this by the result of a cluster analysis of the UK Wealth and Assets Survey's most recent wave. This study is based on a sample of some 19,000 households in Great Britain excluding Northern Ireland, Scotland North of the Caledonian Canal, and the Isles of Scilly. The survey has very extensive data in detail on forms of wealth and income for households rather than individuals. This is important because in contemporary post-industrial societies given the significance of multiple—usually couple—earners in households, it is the household which is the most significant unit for the possession of the kind of resources which constitute class in Weber's sense, that is to say the resources which can be used in market relationships. Moreover, the data includes detailed measurements of wealth in terms of the overall components of physical wealth, property wealth, financial wealth and pension pot assets, not just in terms of these broad categories but also of sub-components within them, and detailed accounts of the sources of household income. This means that it is not only possible to construct classifications based on the simple possession of income and wealth in relation to market power- the Weberian frame—but also to explore sources of “unearned” income in the form of rent from property (as opposed to simple owner occupation) and income derived from financials assets. That is to say the data gives us a view of the possession of assets which represent command over the labor power/wage incomes of others and thereby allow a first order account of distinctions in the Marxist frame. Despite considerable efforts made in the design and management of the survey data, particularly through weighting and imputation, survey data sets of this kind are generally understood to under-represent households with very large assets. They do not address at all the assets held by UK citizens and members of their households which are located in other tax regimes and much of the beneficial ownership of very large wealth is located in tax havens e.g., Monaco. Neither do they address the way in which very wealthy families use discretionary trusts to avoid taxation^1^.

## Table One Near Here

Table One shows the pattern of wealth by forms and total and incomes generated by a hierarchical fusion clustering approach using wealth and income components as the classifying variables^2^. This clustering generated five interesting clusters which were sufficiently different. Table One shows the characteristics of the clusters in terms of the variable values used to construct them and of the medians for each component of income and wealth. Both wealth and income are unequally distributed but wealth is much more unequal than income with just over 10% of households holding more than 40% of wealth and with the least wealthy nearly 60% of households holding <20% of wealth.

Table One also shows how wealth and income are related in relation to the categories of each. The high wealth and income and very high wealth and income clusters (just over 10% of cases) hold more than 55% of financial wealth, more than 40% of pension wealth and nearly 40% of property wealth whereas the bottom two clusters (nearly 60% of cases) hold <10% of financial wealth, <15% of pension wealth, and <20% of property wealth. The top two clusters containing just over 10% of households receive more than 60% of unearned income from investment and rent and more than 40% of private and occupational pension income. The middle cluster receives income and holds wealth somewhat above its proportion in the population but at nothing like the scale for the high and very high clusters. Table Two shows the forms of wealth held by the respective clusters as percentages of the wealth held by those clusters. What is particularly notable is the importance of financial wealth as a form of wealth for the very wealthy cluster where it is of the same order as property and pension wealth whereas these categories far exceed financial wealth for all other clusters.

## Table Two Near Here

In quantitative in investigations which can inform our understanding of class two key issues are what we measure about our cases and the nature of the cases for which those measurements are made. The review of household level data covering both income and wealth in relation to the forms of both enables us to say something which relates to both of the real dominant framings of class—Weberian in terms of life chances and Marxist in terms of control over the labor power of others. This is in marked contrast to the construction of classes as what purports to be a class theory on the basis of occupational data for individuals. Table Three shows that the relationship between individual occupational class and household income and wealth is not particularly strong. The Spearman R2 for the relationship between occupational category and cluster is just 0.21 i.e., the relationship accounts for about 20% of the variation. More importantly the lowest cluster by wealth and income contains nearly seven times as many respondents or partners who are in managerial or professional occupations as the very high income cluster and nearly 80% as many as in the high income cluster. When we are interested in inequality as a basis for class in post- industrial capitalism occupational categories are not particularly useful.

The quantitative exploration—note that word exploration—of class in post-industrial capitalism is primarily about the actual data but we also have to pay attention to the statistical methods deployed in research. Let me be blunt. Conventional regression based models which seek to assign causality in relation to class position, primarily the position of individuals but sometimes of households, are as much use as a chocolate fireguard. This applied not only to basic regressions but also to complex econometric style developments of regression. The fundamental problem is the ontologically invalid reification of variables outwith the cases themselves. There are alternatives and Ragin and Fiss (2017) examination of *Intersectional Inequality* using the figurational technique of Qualitative Comparative Analysis (QCA) which allows for both complex and multiple (equifinal) causation is a particularly interesting study which should be replicated using other data sets. Another approach which was the basis of analysis of “The Great British Class Survey” (Savage et al., 2013) is Latent Class Analysis, a development of Factor Analytical approaches which permits the deployment of non-scalar data. This is different from cluster analyses approaches, particularly hierarchical clustering which generated the clusters described in this paper. Hierarchical clustering is an agglomerative approach in which cases are compared to find the most similar pair which are then conjoined and resulting clusters are constructed in sequence until all cases are in the same cluster. The method is one based on comparison. It starts from the characteristics of cases rather, than the approach of Latent Class Analysis which assumes that the measured variables indicate the existence of an underlying set of categories for a categorical variable.

Latent Class Analysis, like Factor Analysis, is a variable centered method. Agglomerative fusion is a good method for as Tukey (1977) prescribed: seeing what the data are telling us.

In all work exploring class it is essential to make a heuristic distinction between class position and class identity. The first is a relational description of cases of whatever form which is best made in relation to an clear conception of the underlying generative mechanisms which express class in the actual social world and which we can measure empirically. Class identity is what people think about themselves. This is formed by social relations as expressed in class position but is engendered by the agentic reflection of the person framing their own identity in time. Quantitative survey work can be used to explore class identities as well as class positions. We can ask people about these things and should do so. Of course both the way in which we articulate a framing of class in relation to topics covered in a survey and the actual detailed form of both the questions asked and the coding scheme deployed in relation to those questions shape the data generated in a survey. The “Great British Class Survey” used Bourdieu's conception of capitals as things possessed by individuals and interacting in locating the class position of individuals to frame the study. Lui(2015, p. 484) commented accurately: “… when we turn to examine the class model proposed by Savage et al. it looks very much like their descriptions of the seven classes are mere reiteration of their operationalization procedures.” Capitals were defined as measured variables without, as Lui notes, any understanding of this variation as engendered by social structural forces. There was no real sense of generative mechanism, not even through a deployment of Bourdieu's own way of handling this through the notion of fields.

Individuals and households are not the only cases relevant to class which can be explored quantitatively. It is useful to look at spatial units in class terms and to construct socio-spatial mappings of class by area. In England this can be done using census data for Super Output areas. There are also spatial data sets available which describe small areas by various formulations of the Index of Multiple Deprivation which can serve as a useful proxy in exploration of how classes are located in space. These techniques can be used at any spatial level but are perhaps most valuable when used to describe the spatial distribution of class within city regions since the city region is a particularly meaningful spatial unit for so many aspects of social life. Usually the data which is aggregated is from censuses and based on occupationally defined class. The only data about wealth which is available at these spatial aggregate levels relates to housing tenure, form and numbers of bedrooms along with data about car ownership by households. There is very limited data available which describes income at small area levels, usually only if there has been an extensive local social survey for administrative purposes. Social Geography studies typically explore variation through space. An excellent example is (Hamnett, 2003) *Unequal City* which examined change in the socio-spatial structure of London over a 40 year time span. The existence of small area style census data means that the spatial aspects of factors relevant to an understating of class can be described dynamically. We can see how things change. In the UK one aspect of such change is the radical shift in the relative social location of the populations of social housing areas, particularly of the very large council housing estates built in the years from the end of the second world war up to the 1970s. These estates, particularly but not exclusively the housing in them built as semi-detached villas in quasi garden city layouts, were occupied by household which were “respectable”—a crucial expression describing the cultural and reputational aspects of class position in an industrial social order, The combination of “right to buy” by existing tenants^3^ and more importantly the deindustrialization of industrial city regions (to an extreme extent in London) has led to the dispossession and marginalization of these areas. In the twenty-first century post-industrial UK the “good life” [for the working class] as lived in the third quarter of the twentieth Century' is now found in speculatively built owner occupied estates. Many of the residents of such locales are the children of the council tenants of the earlier period (Banim, 1986; McEwan, 2018). This spatial change matters and quantitative dynamic trajectories describe it.

The best way to sum up the value of quantitative, and especially quantitative descriptive, work in relation to class is to return to Williams assertion that the industrial word was unknowable without counting. So is the post-industrial world. To say this is not to privilege the quantitative over the qualitative but rather to recognize that quantitative description, especially description of change, is a way both of setting the context for intensive investigative qualitative investigation and provides a basis for generalizing claims for the findings of ideographic studies. To understand class we need to both count and explore in qualitative mode. To that assertion this paper will return.

## The Role of Qualitative Research in Understanding Class

Class has to be understood, whatever mode we employ in developing that understanding, in dynamic terms and as an emergent phenomenon at every level of its manifestation. That means that narratives, accounts of how things develop through time, are central to any understanding. The use of data series to describe change through time is no less a narrative than any textual account. Here the focus is on such textual accounts beginning with the role of historical methods developed as historical sociology/sociological history. E.P Thompson's two great books *The Making of the English Working Class* and *Whigs and Hunters* provide a model for the kind of history of classes in creation (*The Making*) and under assault (*Whigs and Hunters*). In the era of the *Unmaking* of the old industrial working class historical studies have a crucial role to play. One very important point to make here is that the role of the state in many forms and at many levels is crucial to any historical account of these processes. The creation of disciplinary silos in academic work means that sociological discussions of class are very poorly informed by reference to or understanding of the role of benefit systems in relation to both class location and class experience. Thompson very well-understood the significance of the New Poor Law of 1834. The investigators associated with the Great British Class Survey paid no attention to the emerging significance of Universal Credit in the twenty-first century UK for households which contained paid workers. Likewise urban planning policies which subordinate local government to the logic of a secondary circuit of accumulation and the interests of speculative development capitalists—capitalists because agency matters (Fitch, 1993) are important not only in terms of the constitution of space. Labor and Social Democratic parties, the political representatives of the industrial working class, have embraced a “necessary pragmatism” and abandoned the interests of the class which founded them, We need histories which incorporate state actions and note the role of political actors.

And we need histories which address the role of the collective organizations of the working class, Labor and Social Democratic parties, trade unions and cooperatives not just in terms of the formal institutional development and often transformation, but also with a focus on how they relate to the lived experience of class on a daily basis. One of the most depressing aspects of the way class is lived after industry is the extent to which such organizations have a much reduced (trade unions and cooperatives) or even antagonistic (most European Social Democratic parties) relationship with how people live class. In the era of austerity the role of formerly class oriented parties in governance has been almost wholly negative for many people. The key elements in this have been the subordination of municipal governments to capitalist developers and the privatization/quasi privatization of public services, notably social housing. The way in which English social housing has been transferred to quasi private Arms Length Management Organizations (ALMOs), often against substantial resistance by tenants illustrates this very well. Local histories of organizations like Gentoo which took over the housing operations of Sunderland Council and now manages nearly 30.000 homes are absolutely necessary to framing a historical formulation of class experience now. The actual character of local governance and in particular the organizations identified by Stewart (1994) as components of the new magistracy play a crucial role in how the state relates to people in class terms. These organizations, for example in the UK Local Enterprise Partnerships (LEPs), give elements of the bourgeoisie a crucial role in areas where they could not be elected public night soil remover in a democratic contest. Sometimes these people are real industrial capitalists but more often now they come from real estate capital or the lawyers, consultants and financial professionals who service capital. Senior public managers from quasi privatized public sector bodies, notably in education, are also present. If we are to understand class we have to understand classes in the plural and these are locales in which elites are to be found. We need to identify and describe power elites at every level. Here Mills (1956) approach still works but we can also use techniques like social network analysis to refine the way in which relationships exist. The inter-linkages can be identified not just from official documents but also from press coverage^4^. Here the necessary qualitative skills are those of documentary analysis and interpretation.

There is an enormous amount of documentation, most current material online, which gives an insight into how governance works. The tools of the historian are entirely appropriate but there are particular practical tricks which are worth learning, although the only way to really get to grips with this material as with any qualitative work is to dive in and engage. A good starting point is always the minutes of meetings when researching a specific issue. Minutes are supported by documents referenced in them and sometime these are not publicly available at first try. Documents which are held back from public view are always especially interesting. Researchers need to know how to use Freedom of Information (FOI) requests and how to be persistent in pursuing these. The cover often deployed to circumvent releasing material in the UK is commercial confidentiality. This should always be challenged but the very assertion of it is indicative of class relations and connections. Campaign groups, for example groups campaigning about planning decisions, are often both skilled in these forms of research and well-informed about issues which matter. Groups, entirely properly, are concerned with specific issues (although planning issues are often general in their implication) so if researchers investigating how class interests of elites are enacted at the level of the locality/city region need to be able to synthesize across a range of issues to develop a comprehensive account of what is going on. In the UK at least the traditional role of the local media in scrutinizing local governance has been almost completely abandoned. The coverage even of the operations of elected local bodies is minimal and the only way in which the unelected governance agencies ever appear in the media is on the basis of their own press releases, although even these can be useful as a starting point. Investigating the local state in all its forms is crucial for class focused research and is not done anything like enough, particularly as the basis of Ph.D. studies.

On reflecting on this discussion of the importance of historical investigation of the state at all levels, it is evident that the experiences which form class locations and identities are not just experiences which derive from work. Throughout modernity, and to an increasing degree in late modernity, relationships with the state in all its forms also have constitutive power. That said work and work relations also matter and here historical work, including the collections of oral testimonies either describing individual life courses or more collective forms of historical construction, have an important role to play. Examples of collective construction of experience include (Byrne and Doyle, 2005) use of focus groups stimulated by visual elicitation to reflect on the meaning of the coal industry and its termination in South Shields and (Warren, 2018) similar deployment of the method on Teesside. Warren's study was particularly interesting because many of his informants were reflecting on their work experience in relation to life experience in what might be considered as a paradigm of organized capital, ICI. Moreover, this study incorporated the experiences of women, and very interestingly women who were part of the significant in status terms industrial middle class. The industrial middle class, the staff as opposed to the shop floor, were a crucial component of the class structure of industrial capitalism. Historical work on class has to address their experiences too.

All the techniques of qualitative work are valid in investigating and understanding class. These techniques are best understood as fuzzy sets which intersect with each other. So qualitative interviews can be used to explore all aspects of class, not only in relation to situating respondents themselves in terms of local and identity now and through their life courses but also as sources of information about other people and as descriptions of institutional and political forms and processes. Ethnographic observations, at whatever location on the continuum from complete observer to complete participant, construct descriptions of class as it is lived in action in all ways. For example participant observation in planning inquiries (although often closer to complete observer role) yields very valuable information not only about the formal outcomes of such processes but of the way powerful actors, both individual and institutional, relate to and interact with each other. Any open venue of governance, but particularly those in which there are disputes informed by evidence, lets the researcher see something of how class is lived as power by the powerful. One form of ethnographic work which was very evident in the industrial era is participant observation in work contexts. This has not gone away altogether, and remains a very important and useful mode, but such ethnographies, particularly participant ethnography by those who themselves work in the context being studied, are now more likely to come from health contexts than from manufacturing. This is not to devalue them at all. Health work is one of the most important forms of work in post-industrial capitalism and ethnographies of how class is lived in that kind of work are enormously useful.

There are a number of ethnographic or quasi-ethnographic- a term used to describe studies which whilst including an element of direct observation are also reliant on interviews—of work in a range of post-industrial contexts. Examples include (Warren, 2011) exploration of “Living the Call Centre,” and a number of studies carried out within the frame of reference of what Burawoy has called “the extended case study,” for example (O'Riain, 2000) Ethnographic investigation of how work hierarchies actually play out in terms of lived experience is crucial to understanding class at any time and in any place.

Likewise ethnography of all lived experience, buttressed by whatever complementary approaches are deployed (e.g., interviews), is not only legitimate but necessary as a mode of understanding class. In post-industrial and post-welfare capitalism ethnographies of how people relate to the state in terms of any aspect of state provision, but in particular in relation to the operation of income maintenance systems, tell us a great deal about what class is and how it is lived. The ways in which people experience the different modes of conditionality attached to the receipt of Universal Credit in the UK are ways in which class is lived and the more we know about them the better. Ethnographies necessarily include representations in modes outwith that of traditional academic writing. So novels, plays, poetry songs, films, cartoons, any form of representation, is valid and informative. Ken Loach's films *I, Daniel Blake* and *Sorry We Missed You* are ethnographies of living on benefits and working in the gig economy. They have rightly been praised as Art but they are more than Art—they are representations of how class is lived. This is in marked contrast to reality TV Poverty porn, exploitative, selective, and deeply offensive. Richard Dawson's songs and music stand at the other extreme from that exploitative rubbish—pointing up for example the intersections between class and disability as he has lived them. This is life history as music.

Here a cautionary note is appropriate. Many ethnographies of class have focused on what Byrne (2005a) called the “dispossessed working class.” But important though the dispossessed are their experiences do not constitute the whole set of working class experience in post-industrial capitalism. There are more who belong to what Byrne (2005b)called “the missing middle,” people living in households in the middle of the income range, in the UK owner occupiers although usually not outright owners, with now at least secondary and often University levels of educational qualification, and often working in semi-professional or even professional roles. Many workers in education, health, the emergency services and so on, along with IT workers and what remains of the highly skilled technical working class, for example instrument artificers—tiffies—the key skilled trade of many process industries, belong to this category. There is a tendency in working class studies to look only at those who have been absolutely immiserated, particularly in work done in the United States. The US is an outlier. Its working class has never had any real direct political power, was always smaller proportionately than European working classes, was much weaker in terms of cultural class identity, and was uniquely affected by the continuing significance of racial division based on a history of chattel slavery and miscegenation laws^5^. This is not to say that ethnicity does not always matter in relation to class and that there is not racism in other post-industrial societies but the US is a special case. In many ways the UK stands at the other extreme, not least because by far the largest post-colonial population is the invisible minority of the Irish descended who in any event have been massively inter-married with English, Welsh and Scottish people for at least 200 years. It is notable that inter-marriage between “Brits” and those immigrant post-colonial populations “of colour” who arrived in the industrial era is very common indeed.

## Studies in and of Places – Mixing Up Methods

To understand class we need more than one method. To say this is not to insist that every individual study is multi/mixed method, although there are always advantages in going that route if possible. However, social scientists should always embed their own investigations both in design and in interpretation in relation to the broad range of work dealing with their topic of interest. The scientistic format of the literature review serving only the purpose of detailing the findings of existing work so as to identify the new contribution of this work is not appropriate for us. We need to engage in meta interpretation—to draw in an explicitly synthetic fashion on as much material as we possible can and enter into a dialogue with it in the exposition of the actual specific research exercise we have carried out. Beyond this there is an absolute necessity for synthesizing scholarship, not based directly on any research exercise but drawing on as many studies as possible in relation to explicit theoretical framingS (the plural needs emphasizing).

One set of studies provides an example of how research of this kind can be done in a multi- disciplinary and mixed method style. Class is lived in places. The ways in which class is lived at work, in domestic life, in relation to the state, all play out in spatial locations. People move around in space at every spatial level up to an including trans-oceanic migration. However, there is a particular value to studies which look at class as lived and class as a set of relations in particular places. There is a role in understanding class for what Sociology and Social Anthropology call “Community Studies” and Social Geography calls “Locality Studies.” The usual spatial range of the latter in the urbanized world of post- industrial capitalism should be something like a city region as was done by (Beyon et al., 1994) and Byrne (1989). That range is big enough to explore most but not all of the relational processed which determine (in Williams, 1980 sense of that word determine as setting limits rather than exact specification) both class location and class identity. Plainly there are social forces which operate beyond the spatial level of the city region in the form of trans-national and finance capital but even they are expressed in and through lived experience on the scale of the locality. To explore how class is lived at this scale it is absolutely necessary to deploy a mixed methods approach to investigation. The best way to explain what this implies is by taking a real example and working through how class can be investigated in that place.

My example is the Leicester city region in East Midlands of England. The consists of the City of Leicester, Leicestershire, and Rutland and has a population of just over one million. There are three first tier local government entities—Leicestershire County Council with a population of 650 thousand distributed across seven district councils, Leicester City itself with 330 thousand people and Rutland with 37 thousand people. The local health economy—a very important term in relation to the governance of the UK National Health Service (NHS)– is managed in a variety of ways but of crucial significance are the clinical commissioning groups with three covering the city and two parts of the county plus Rutland. This part of the East Midlands was very heavily industrialized with coal mining, textiles especially in knitwear, shoe manufacture and a range of engineering plants. It is particularly interesting because it is very multi-ethnic, at least in Leicester City itself, with a large South Asian population primarily of Gujerati background coming from East Africa and Gujerat itself. There are also significant Afro-Caribbean, African (including Somali), and European accession state populations, particularly Polish. My former partner lives in this city region and my younger daughter is Leicester girl born and bred and lived there until going to University.

This area is very much deindustrialized. Just over 12% of jobs are in manufacturing and all coal mines have closed. In 1981 over 45% of workers were employed in manufacturing and mining. There was a very substantial female industrial labor force. The ethnic composition of Leicester City in 2011 was 45% identifying as White British, 5% as White Other, 28% as Indian, and 21% as other ethnicities including Afro-Caribbean, African, and Pakistani. For the County area the figures were 89% White British, 3% White Other, 4% Indian, and 4% all other ethnic groups. The 1981 Census did not record self identified ethnicity but rather country of birth. Then in Leicester City 82% of the population were UK born and 13% were born in East Africa or India. In 1981 Leicester was still very much industrial and it is important to note that many of the Indian population were engaged in industrial work, particularly in knitwear. This was also true of the Afro-Caribbean population but the African population arrived “after industry” although the EU accession state migrants often do work in what remains of the city region's industrial base. Plainly any study of how class has been and is lived in this area has to take account of the intersectionality of all of class, ethnicity, and gender. Moreover, that complex intersectionality has itself been dynamic over time.

Already I have started to use data series derived from the decennial censuses and other sources to provide a quantitative narrative of this place. Other data which would be relevant includes patterns of housing tenure, patterns of residential location, educational backgrounds, and information on the detailed patterns of employment in the post-industrial service centered economy. Using available secondary data sources all of these can be mapped through time, across quite fine grained spatial locations, and to a considerable degree across time at that fine grained level. Aggregate spatial data is enormously useful in providing trajectories of differentiation. It is possible using local election results to provide a somewhat coarser but still useful account through time of class as expressed in political preferences, although the low turn out in UK local elections means this might often be about how class is not expressed. Note that almost all this data takes the form of spatial aggregates. Micro data might be available from dedicated surveys or, possibly with considerable and reasonable difficulties of access, from administrative data sets. However, the aggregate data very much helps in telling the story. In an ideal world it would be possible to commission a large scale sample social survey investigating elements of both class position and class identity—and do this in with reference to multiple framings of both. This would take the form of a more extensively informed—that is informed by reference beyond a single class framing—local version of the Great British Class Survey, although ideally this would be a survey based on a household case base rather than simply collecting data from individuals.

When we turn to the qualitative the starting point is historical work on the documents. Here the terms “document” needs to be understood as broad and general. For example images, both still and moving, form part of the documentary record. In principle the most easily accessible documents are documents produced as part of the processes of governance. The activities of the state are recorded and these records include important sets of administrative documentation describing the actual way in which things are done. Certainly any attempt to grasp how the state has played a constitutive role in the class position of working class people will necessarily involve a careful review of the history of social housing as represented in the documentary record of the local authority and other housing bodies. Of course the paper (although often now web based) trail is by no means the whole story. There is a necessary role for interviews—both of political and administrative actors and of local residents (where focus group discussions can be very productive)—as a means of generating an oral history to set against the documentary record. The same combination of documentary based investigation and the generation of oral testimonies can be deployed in constructing other historical records. These might include a history of planning and development policy in association with local industrial and enterprise support policies, an area which can show the character of local power elites and how they seek to influence policy in their own class interest. In relation to the class aspects of austerity and the role of the new public sector management in relation to workers' experience, policy developments in the health, and education systems are of considerable interest. Archives can be accessed to examine local industrial histories, for example of GEC which was enormously significant in the Leicester Urban area but also of the knitwear industry. No single researcher or even research project will have the time and resources to cover all these fields and materials but an important skill is the competent meta interpretation of secondary sources. Use other people's work, ideally discussing your interpretation of their findings with them, in synthesizing a general historical narrative.

That kind of work is retrospective and when it moves into causal accounts retroductive. How can we look forward to see how class relations will develop and class will be lived in the future? We might use data about a place as the basis of a simulation to project possible futures in which we use different values of key control parameters to see what different futures might emerge. The approach developed by Allen (1997) could be adapted as a basis for simulations of this kind. Such simulations have a political potential because they enable us to show how the adoption of different policy positions would have consequences for the future socio-economic-ecological^6^- political context. Probably the most useful approach to envisaging futures is to ask people and focus groups are a particularly good method for doing this. (Tukey, 1977) asked children in Dijon and York to envisage the futures of the places in which they lived. This approach can easily be adapted to explore how people see their futures in relation to living class and seems particularly appropriate as a method to deploy with young adults who will live that future.

## Conclusion

Let me return to the crucial assertion made in the abstract to this article. Social science as an empirical (not empiricist!) programme is always about developing knowledge on the basis of the interaction of theoretical framing and empirical investigation. Merely empirical work, like much of that conducted using trivial linear methods by the Nuffield school, tells us almost nothing worth knowing about class as it is and as it is lived. Abstracted theoretical assertion by victims of what Thompson called “the French flu”^7^ is if anything even more pointless and offers nothing to any programme of class based radical social change. What has been attempted in this piece is a review of how we can do research on class in ways which integrates theoretical framing—which plainly for the present author is based on Marx's original conception as that has been developed by others over nearly 200 years—and investigation of how people live class, how the class structure determines—(again in Williams, 1980 sense of setting boundaries to possibilities)—they live class, how they understand class as they live it, and how social structures, institutions and above all relational class affects all of these. Above all else without that kind of understanding we as social scientists will not have much to say to the emancipatory programmes of class politics. If there is one point which I would assert as strongly as possible in this conclusion it is that whilst work relations continue to be enormously important in relation to class, so now are relations with the reproductive aspects of capitalism as these are manifested, largely through agencies of the state. This matters in relation to experience since so much of life is actually lived in relation to and through administrative and institutional structures which are part of state system. It matters in relation to how class works out in terms of power. That is why it is essential to explore the way in which elites function. Political history does this primarily at the level of national states and policy formation. That level has direct effects particularly through income maintenance systems which in most post-industrial states are national functions. But what matters is not just policy formation but also implementation. It is at the level of the city region that we an explore the intersection of policy and implementation Local governance, and the bodies of the new magistracy in the form of unelected state agencies such as Local Economic Partnerships, are just as important here as elected local government itself. Likewise in relation to how health is lived health systems are enormously important, not just as major employers, but as major determinants of how life is lived and how it is experience. The state matters in all its forms and it is often through examination of the local state forms that we can see the relationships between class and power. And understanding must address the dynamism of every aspect of class at every level in relation to all the complex systems with which class relations are interwoven^8^.

## Data Availability Statement

Publicly available datasets were analyzed in this study. This data can be found here: https://beta.ukdataservice.ac.uk/datacatalogue/series/series?id=2000056.

## Author Contributions

The author confirms being the sole contributor of this work and has approved it for publication.

## Conflict of Interest

The author declares that the research was conducted in the absence of any commercial or financial relationships that could be construed as a potential conflict of interest. The handling editor declared a shared affiliation, though no other collaboration, with the author at time of review.

## Footnotes

^1^National Accounts aggregate data generally gives totals for wealth which exceed that produced by aggregating sums from the micro household level data generated by surveys of the kind of the Wealth and Assets survey. There is a literature on this issue of coverage which is summarized in Chakraborty et al. (2019) who ask the question: Is the Top Tail of the Wealth Distribution the Missing Link between the Household Finance and Consumption Survey and National Accounts?

^2^The indicators chosen were simply Gross Household Income and Total Household Wealth as defined by ONS together with the percentages of the value of wealth in the forms of physical, financial, property and pension AND the of the forms of income as earned (employment and self-employment), unearned (rents and financial asset incomes), pensions and benefits held and received by the households in the sample as cases. The percentages were percentages of the total value of wealth and income for each element rather than percentages of wealth and income as held by each case. Since these are monetary measures at a scalar level their operationalization is a function of the mode of data collection and the simple arithmetic division used to construct them from the monetary measures.

^3^Over the years the sale of properties purchased under right to buy, usually to divide an asset among the children of the purchasers, for whom the creation of an asset to pass on was an important motive in purchase, has transferred much, in London most, of these dwellings to private landlords who bought to let.

^4^Award ceremonies are particularly interesting here. In the past linkages could be established posthumously by attendance at Masonic funerals. Now look who is giving each other awards at black tie dos.

^5^Brazil is the other great, although not only, post chattel slavery society in the Americas and the world. The one drop of blood rule does not seem to have had the same salience in Brazil where economic location was very intersectional with ethnic background.

^6^Many of the simulation models for this kind of work are located in the ecological literature. In a context of climate crisis incorporating an ecological component into Modeling is essential.

^7^And dealt what should have been a death blow to that style of work in *The Poverty of Theory* (1978).

^8^Including of course intersectionality with gender and ethnicity although both kinds of intersection are very much context dependent. This is not just a matter of national level differences. Local differences can be profoundly significant. Leicester and Bradford both have very large South Asian originating populations but the interaction of locale of origin, specific ethno-religious identity, and industrial structure means that the intersections of ethnicity, gender, and class work out very differently in these places.
